# IMPLEMENTING AGE-FRIENDLY HEALTH SYSTEMS: SCALING 4MS CARE ACROSS VA

**DOI:** 10.1093/geroni/igad104.2985

**Published:** 2023-12-21

**Authors:** Laurence Solberg, Shivani Jindal, Kimberly Church, Andrea Schwartz

**Affiliations:** NF/SG VHS, Newberry, Florida, United States; VA Boston Healthcare System, Boston, Massachusetts, United States; VA Central Office, Washington, District of Columbia, United States; VA Boston Healthcare System, Boston, Massachusetts, United States

## Abstract

The U.S. Department of Veterans Affairs (VA) provides health care to 9 million enrolled Veterans across a variety of settings. Nearly 50% of enrolled Veterans are age 65 and older, compared to 16% of the U.S. population. The VA has set out to become the largest integrated Age-Friendly Health System (AFHS), using the 4Ms as a framework to improve care for older adults: what Matters, Medication, Mentation and Mobility. When implemented together, the 4Ms allow interprofessional teams and health systems to provide care consistent with what Matters to each older adult. Since joining AFHS movement in 2020, over 190 care settings at 100 VA facilities have earned recognition from the Institute for Healthcare Improvement (IHI). VA has identified key drivers to scale-up and spread 4Ms care at the national, local, and team level. Drivers include active and visible national leadership, building a coalition of local champions, and regular commmunication with teams. VA has accelerated adoption of the 4Ms by following IHI’s Action Community (AC) model. At close of registration, 145 teams from 69 VA facilities enrolled in the VA AC. The VA AC offered a series of monthly webinars and coaching calls to empower teams to create a 4Ms plan building on exisiting best practices. In addition to using evidence-based tools identified by IHI, teams may also leverage VA clinical innovations to support their 4Ms plan. Implementation of AFHS provides a framework for interdisciplinary teams to continuously improve Veteran-centered care and ultimately, fulfill VA’s mission to honor America’s aging Veterans.

